# Invasive listeriosis in Finland: surveillance and cluster investigations, 2011–2021

**DOI:** 10.1017/S0950268823001073

**Published:** 2023-07-10

**Authors:** Kristiina Suominen, Sari Jaakola, Saara Salmenlinna, Maria Simola, Suvi Wallgren, Marjaana Hakkinen, Annika Suokorpi, Ruska Rimhanen-Finne

**Affiliations:** 1 Department of Health Security, Finnish Institute for Health and Welfare, Helsinki, Finland; 2 Finnish Food Authority, Helsinki, Finland

**Keywords:** listeria, food-borne infections, surveillance, microbiology

## Abstract

Foodborne pathogen *Listeria monocytogenes* may cause serious, life-threatening disease in susceptible persons. We combined data from Finnish national listeriosis surveillance, patient interview responses, and laboratory data of patient samples and compared them to listeria findings from food and food production plants collected as part of outbreak investigations during 2011–2021. The incidence of invasive listeriosis in Finland (1.3/100000 in 2021) is higher than the EU average (0.5/100000 in 2021), and most cases are observed in the elderly with a predisposing condition. Many cases reported consuming high-risk foods as well as improper food storage. Since ongoing patient interviews and whole genome sequencing were introduced, several listeriosis outbreaks were detected and food sources identified. Recommendations about high-risk foods for listeriosis and proper food storage should be better communicated to susceptible people. In Finland, patient interviews and typing and comparing listeria isolates in foods and patient samples are crucial in solving outbreaks and determining measures to control invasive listeriosis.

## Introduction

Invasive listeriosis is caused by the foodborne pathogen *Listeria monocytogenes.* It mostly affects people with a predisposing condition, such as immunocompromised persons, the elderly, pregnant women, and neonates, causing sepsis, infections in the central nervous system, stillbirth, and foetal death [1, 2]. The mortality rate can be as high as 30% [3].

*L. monocytogenes* is ubiquitous in nature and can withstand variable environmental conditions, such as temperatures from 2 to 45 degrees Celsius, low pH, and high salinity [2]. It has been shown to persist in food processing plants, causing a significant public health burden and possibly prolonged outbreaks over many years [4].

In 2021, listeriosis was the fifth most reported zoonotic disease in the EU and, along with West Nile virus infections, had the highest mortality rate [5]. In that year, Finland had the highest notification rate of listeriosis in the EU, and during 2017–2021, the annual notification rate was two to four times greater than the EU average [5].

ECDC has envisioned whole genome sequencing (WGS) to be the method of choice for typing of microbial pathogens to improve disease surveillance, outbreak investigation, and the evaluation of prevention policies [6]. In Finland, WGS was introduced in 2015 for listeria confirmation and typing, and interviews of listeriosis cases were launched in 2016 at the Finnish Institute for Health and Welfare (THL). In 2015 and before, pulsed field gel electrophoresis (PFGE) was used for the molecular typing of listeria. We collected surveillance data of listeriosis cases notified to the Finnish Infectious Disease Register (FIDR) in 2011–2021, laboratory data of patient samples, and patient interview data. These data were compared to the listeria findings from food and food production plants collected as part of the outbreak investigations to describe how the change in the typing method affected the listeriosis surveillance, and to describe the risk factors for infection.

## Methods

### Listeriosis surveillance

Since 1995 in Finland, clinicians and clinical microbiology laboratories report data on culture-confirmed invasive listeriosis cases (gender, age, place of residence, caretaking institution, isolation site of the bacteria, and sampling date) to the FIDR and submit *L. monocytogenes* isolates (from normally sterile tissue and from foetus, stillborn, newborn, or the mother) to THL [7, 8]. We formed a database of cases notified to the FIDR between 1 January 2011 and 31 December 2021. We linked the laboratory data of patient samples in 2011–2021 and patient interview data in 2016–2021 with the data from the FIDR, and described the cases according to gender, age, and hospital district. The incidence rate ratios (IRRs) with 95% confidence intervals (CI) were calculated for 5-year age groups. Statistical analyses and data description were conducted using the Stata 17.0 software (StataCorp LLC, USA). With Microsoft Excel (version 2202), a linear line estimator for incidence was fitted using a least squares method.

Depending on the isolation site, the cases notified in 2011–2021 were classified as septic, meningitis, or materno-foetal. Materno-foetal cases include pregnancy-associated listeriosis or listeriosis in a newborn (infection in both the mother and the newborn was counted as a single case with the mother being included in the data set). For cases notified in 2016–2021, data on underlying conditions, medications, and the consumption of risk-associated food products were collected by local healthcare staff or THL from patients or their family members using an online questionnaire. The questionnaire focused on the consumption of ready-to-eat meat products and cold cuts; cold- or hot-smoked, cured, and raw fish products; unpasteurized milk and dairy products; frozen vegetables; and convenience food consumed within two weeks prior to the onset of symptoms. In addition, the questionnaire asked about travelling in Finland or abroad and eating in a restaurant two weeks prior to the onset of symptoms, as well as knowledge of risk foods, the habit of checking the fridge temperature, and the best-before dates of the food products. Mortality data were obtained from the Finnish Population Registry. To estimate the case fatality rate, deaths within 30 days of sampling were considered listeriosis related.

### Monitoring of L. monocytogenes in foods

The local official food control laboratories send *L. monocytogenes* isolates from food samples to the Finnish Food Authority (before 1 January 2019 Finnish Food Safety Authority Evira) strain collection and, when needed, for further characterization and typing [9, 10]. The samples have been collected as part of official sampling carried out by authorities, or as part of food business operator’s Hazard Analysis and Critical Control Points (HACCP) plan. Additionally, samples taken as part of food- or waterborne outbreak investigations are sent to the Finnish Food Authority.

The *L. monocytogenes* isolates sent by the local official food control laboratories to the Finnish Food Authority were further typed using the same methods as in THL. In 2011–2017, the strains were typed using PFGE, and after 2018, using WGS. The WGS typing was done using the same method and the same schemas as in THL.

National surveys to estimate the presence and levels of *L. monocytogenes*, especially in vacuum-packed smoked or cured fish products and ready-to-eat products, have been performed within the national monitoring of zoonoses [11]. In 2012–2014, sliced heat-treated or cold-smoked ready-to-eat meat products and, in 2015–2016, sliced cheeses were monitored for *L. monocytogenes.* Samples were taken from retail randomly. All *L. monocytogenes* isolates detected in these surveys are stored at the national reference laboratory in the Finnish Food Authority.

### Microbiological methods

The detection and isolation of *L. monocytogenes* from patient samples were performed in clinical microbiology laboratories. The isolates were sent to the reference laboratory (THL) for confirmation and further typing. In 2011–2014, the species confirmation and serogroup determination were performed using PCR and molecular typing using PFGE as previously described [12, 13]. In 2015, WGS was introduced for listeria confirmation and typing, and it was used in parallel with conventional methods for one year. Since 2016, WGS has been the only method for species confirmation and molecular typing for listeria.

DNA isolation, library preparation, and sequencing on MiSeq (Illumina Inc.) were performed as earlier described [14]. Ridom SeqSphere+ (Ridom GmbH, Münster, Germany) was used for 5′ and 3′-end quality trimming, assembly using Velvet, and for retrieving serogroup and 7-gene multilocus sequence type (MLST) information. For the determination of serogroups from the sequence data, a Ridom SeqSphere task template based on Doumith et al. [15] and Lee et al. [16] was used. For the determination of 7-gene MLST, a task template based on pubMLST.org was used [17].

For creating a core genome MLST (cgMLST) protocol, the Target Definer function of Ridom SeqSphere+ was used to identify 1503 target loci shared by the *L. monocytogenes* Finland 1998 strain, which was chosen as a reference (Genbank accession no. NC_01747.1.) [18], and 45 other complete genomes obtained from Genbank. Default filter settings were used to define the target loci, and the default thresholds (90% of sequence identity and 100% of full length to the reference genome NC_01747.1.) were used for the gene-by-gene analysis. The presence of at least 90% of core genome targets and an average coverage of at least 30-fold were required for each assembled genome to be included in the analysis.

## Results

### Listeriosis surveillance and case interviews

**Figure 1.**
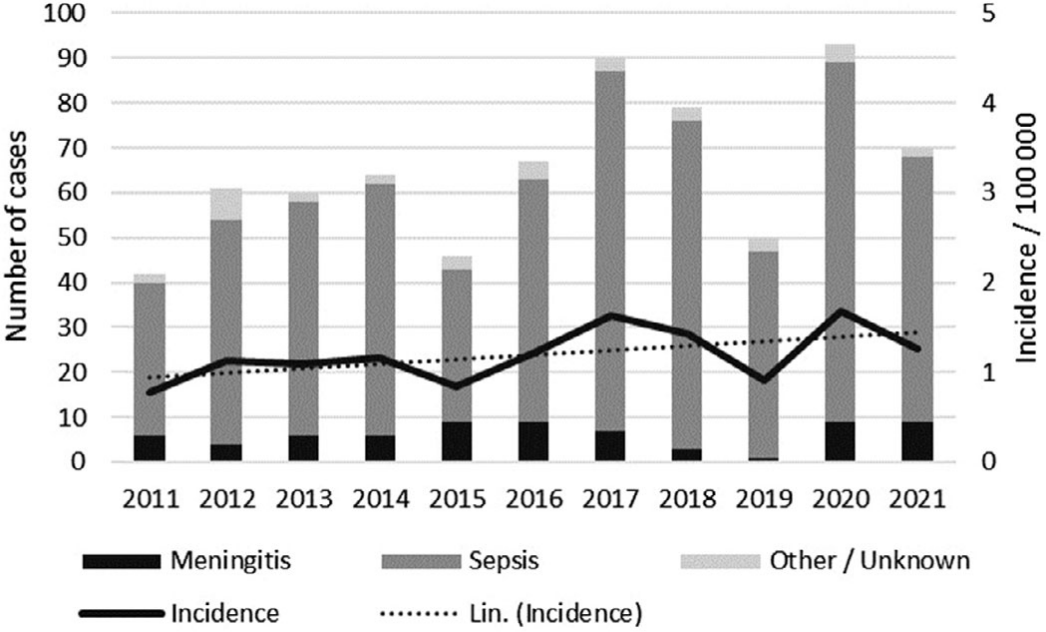
Number of listeriosis cases notified to the FIDR by clinical manifestation, and the annual incidence in 2011–2021 in Finland. A linear line estimator for the incidence is indicated by the dotted line.

In 2011–2021, a total of 722 invasive listeriosis cases (range 42–93 cases/year, annual incidence 0.8–1.7/100000) were notified in Finland (Figure 1). Cases were reported throughout the country, with males and females almost equally represented (48% and 52%, respectively). The median age of cases was 75 years (range 8–101 years), and the incidence rate was 11-fold in those aged >75 years compared to other age groups (IRR 10.6, 95% CI 9.2–12.3). The percentage of septic infections ranged between 74 and 92 per year (Figure 1). Of all cases, 4% (11/722) were materno-foetal. The median age of the mothers was 30 years (range 22–43 years). Serogroups IIa (67%, 487/722) and IVb (22%, 160/722) accounted for most of the cases, while serogroups IIc and IIb caused 6% and 2% of the cases, respectively. The mean annual case fatality rate was 22% (range 13–30%).

Interview data were obtained for 68% (304/449) of the cases in 2016–2021. For 38% (115/304) of the interviewed cases, the person who answered the questionnaire was the patient’s spouse or other relative. Twelve cases (4%) did not have any underlying illness, and of them, four were pregnant women. Of the cases with underlying disease(s), 51% (154/301) had heart disease, 29% (87/301) diabetes, 21% (62/301) other cancer than leukaemia, 20% (60/301) lung disease, 15% (44/301) chronic kidney disease, 13% (40/301) rheumatics, 11% (34/301) gastrointestinal disease, 7% (21/301) chronic liver disease, 7% (20/301) immunological illness, 6% (18/301) leukaemia, 4% (12/301) alcohol addiction, and 1% (4/301) had undergone a tissue transplant. In the last three months prior to listeria infection, immunosuppressive medication had been used by 40% (118/296) of the interviewed cases and medication to prevent acidity of the stomach by 50% (148/298).

**Figure 2.**
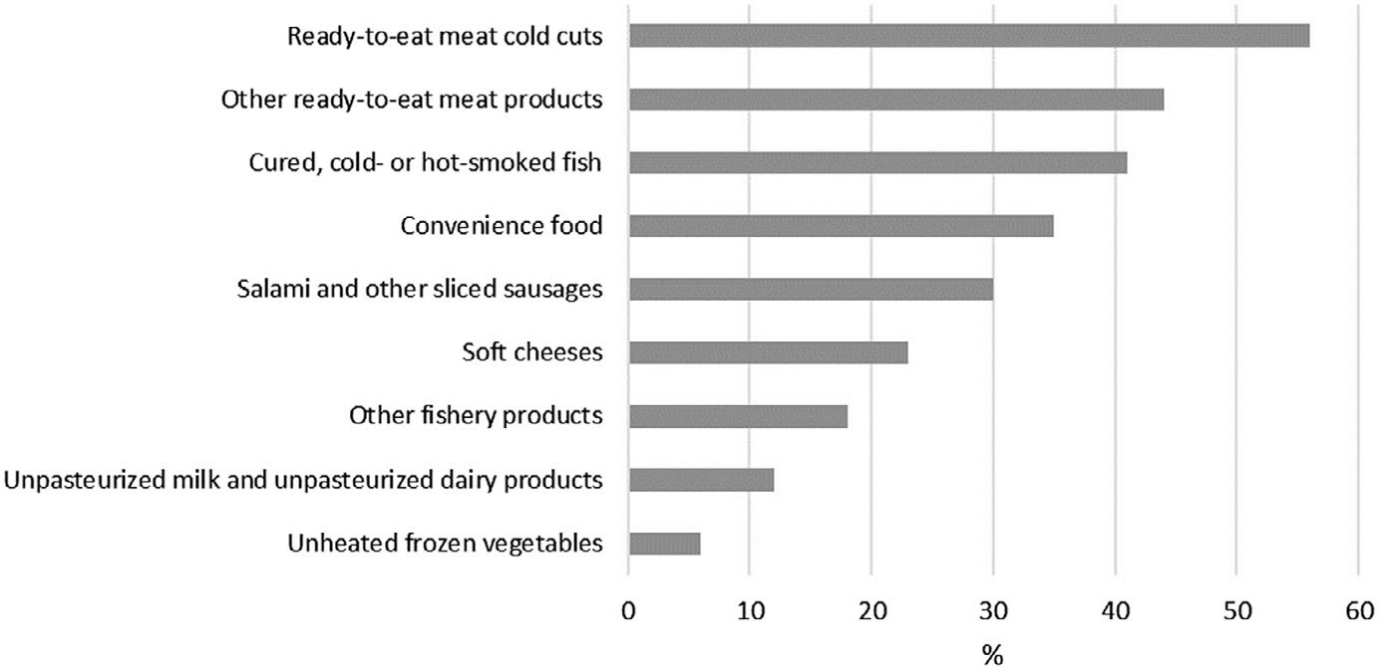
Consumption of risk foods among the interviewed listeriosis patients (N = 304) in Finland in 2016–2021.

Two weeks prior to start of symptoms, 24% (71/297) of the cases had been in inpatient treatment, 29% (84/294) had eaten in a restaurant, 13% (38/285) had travelled in Finland, and 5% (14/283) had travelled abroad. Ready-to-eat meat cold cuts as well as cured and cold- or hot-smoked fish were the most consumed risk foods reported (Figure 2).

Of the cases, 34% (96/286) reported a habit of checking their fridge’s temperature once a week, 9% (25/286) once a month, and 26% (73/286) less frequently, and for 32% (92/286) the information was not available. Furthermore, 63% (179/286) reported throwing out outdated products once a week, 10% (29/286) once a month, and 9% (25/286) less frequently, and for 19% (53/286) the information was not available. Of the cases, 57% (94/166) did not have knowledge of risk foods for listeriosis prior to infection.

Since starting the listeriosis case interviews in 2016, eight listeriosis outbreaks could be solved with both epidemiological and microbiological evidence.

### PFGE and WGS results of patient isolates

**Figure 3.**
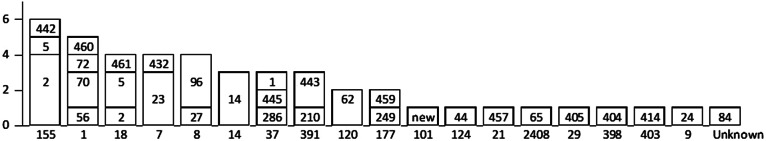
Comparison of MLST (WGS) and PFGE results of *Listeria monocytogenes* in 2015 in Finland. The MLST type by WGS is shown on the x-axis. The PFGE Ascl types corresponding to each MLST type are shown in bars.

In 2015, both PFGE and WGS were used for typing 45 isolates. Eighteen MLST types and 30 PFGE types were identified (Figure 3). Each MLST type contained 1–3 PFGE types. MLST and PFGE were not completely concordant, since two MLST types (ST-155 and ST-18) both contained PFGE types 2 and 5. Three clusters were identified using WGS: ST-155 with five isolates, ST-120 with two isolates, and ST-14 with two isolates. Apart from one isolate, the same clusters were also recognized using PFGE. The ST-155 cluster corresponded to PFGE type Ascl-2, the ST-120 cluster corresponded to PFGE type Ascl-62, and the ST-14 cluster corresponded to PFGE type Ascl-14. One isolate with PFGE type Ascl-5 belonged to the ST-155 cluster in WGS. PCR and WGS were concordant for serogrouping, as all 45 isolates showed same results with both methods.

In 2011–2015, 282 strains were typed and a total of 89 different Ascl profiles were detected in PFGE, the most common being 225, 62, and 96 (26, 18, and 18 cases, respectively) (Table 1). In 2015–2021, 49 different 7-gene MLST types were found from 483 isolates with ST-7, ST-6, and ST-9 being the most common (52, 48, and 41 cases, respectively) (Table 2, Supplementary Table 1).

**Table 1.** Number and origin of PFGE AscI listeria types found in Finland in patient samples (in 2011–2015) and in food and food production plant samples (in 2011–2016). Only PFGE AscI types found in both patient and food/environmental samples are shown

PFGE Ascl profile	Timeline and number of patient isolates	Timeline and number of food/environmental isolates	Categories of food/environmental isolates: (number of isolates in a category)
96	2011–2015: 18	2011–2013: 90	Fishery products (52) Meat products (14) Vegetable products (2) Other food products (2) Fish production plants (15) Meat production plants (3) Food production plants (2)
62	2011–2015: 18	2011–2014: 13	Fishery products (12) Other food products (1): ready-to-eat food
14	2012–2015: 14	2011–2013: 32	Fishery products (30) Meat products (1) Bakery production plants (1)
2	2011–2015: 14	2012–2013: 2	Fishery products (1) Food production plants (1)
5	2011–2015: 8	2011–2013: 6	Fishery products (5) Meat products (1)
23	2011–2015: 6	2011–2012: 3	Meat products (1) Vegetable products (1) Other food products (1): ready-to-eat food
70	2014–2015: 6	2011: 1	Fishery products (1)
72	2011–2015: 6	2011: 1	Fishery products (1)
44	2011–2015: 5	2016: 6	Meat products (6)
84	2011–2015: 5	2011: 2	Other food products (2): ready-to-eat food
24	2011–2015: 3	2011–2015: 10	Meat products (7) Vegetable products (2) Meat production plants (1)
27	2013–2015: 3	2011–2013: 6	Fishery products (4) Vegetable products (1) Meat production plants (1)
21	2011–2014: 3	2011–2013: 3	Fishery products (3)
68	2013: 2	2012–2013: 3	Vegetable products (3)
42	2011: 1	2012: 2	Vegetable products (2)
73	2011: 1	2011: 2	Milk products (2)

**Table 2.** The most common *Listeria monocytogenes* MLST types (with nine or more patient isolates) and clusters (with five or more patient isolates), in Finland in 2015–2021

MLST (Serotype)	Number of patient isolates	Number of food/environmental isolates	Categories of food/environmental isolates: (number of isolates in a category)	Clusters: timeline and number of patient isolates	Cluster contains food isolates: food product
ST-7 (IIa)	52	9	Vegetable products (5) Meat products (1) Fishery products (1) Other products (1) Meat production plants (1)	#1: 2017–2020: 5 #2: 2019–2021: 9^a^ #3: 2020: 12	no yes: chopped iceberg lettuce no
ST-6 (IVb)	48	7	Vegetable products (4) Meat products (2) Other products (1)	#4: 2016–2018: 31^a^ #5: 2020–2021: 14	yes: frozen corn no
ST-9 (IIc)	41	26	Meat products (20) Fishery products (1) Ready-to-eat food (2) Meat production plant (3)	#6: 2016–2021: 27^a^ #7: 2016–2019: 7^a^	yes^b^: frankfurter, meat jelly yes: turkey cold cuts
ST-451 (IIa)	39	19	Meat products (1) Vegetable products (18)	#8: 2016–2021: 6 #9: 2017–2021: 25^a^	no yes: leafy vegetable/green salads, chopped and grated cabbage, chopped leek
ST-8 (IIa)	38	28	Vegetable products (11) Fishery products (16) Meat products (1)	#10: 2015–2021: 6 #11: 2017–2018: 5	yes: frozen vegetables no
ST-37 (IIa)	37	26	Meat products (14) Fishery products (1) Vegetable products (10) Ready-to-eat food (1)	#12: 2015–2019: 6 #13: 2016–2020: 14^a^	no yes: meat jelly
ST-155 (IIa)	24	10	Fishery products (4) Meat products (4) Ready-to-eat food (2)	#14: 2015–2020: 10 #15: 2016–2020: 9^a^	yes: 4 samples from the patient’s fridge (potato salad, frankfurter, steamed potatoes, meat jelly) yes: cured and cold-smoked salmon
ST-1 (IVb)	23	4	Meat products (2) Vegetable products (2)	#16: 2015–2018: 6	no
ST-120 (IIa)	22	1	Meat products (1)	#17: 2015–2021: 20	no
ST-18 (IIa)	16	7	Meat products (1) Vegetable products (1) Meat production plants (5)	NA^c^	
ST-4 (IVb)	14	2	Vegetable products (2)	#18: 2018–2021: 6	no
ST-14 (IIa)	11	2	Meat products (1) Fishery products (1)	#19: 2015–2018: 6	yes: meat jelly
ST-206 (IIa)	12	2	Fishery products (2)	#20: 2016–2021: 12	no
ST-2408 (IVb)	11	2	Fishery products (2)	#21: 2015–2021: 11^a^	yes: frozen rainbow trout fillet
ST-91 (IIa)	11	3	Vegetable products (3)	#22: 2018–2021: 10	no
ST-391 (IIa)	9	2	Meat products (2)	#23: 2015–2021: 5	yes: meat jelly, sliced broiler fillet

a The cluster was solved with microbiological and epidemiological evidence.

b The cluster contains isolates from food production environment as well.

c NA, no clusters with five or more patient isolates.

### L. monocytogenes in foods

In 2011–2021, the Finnish Food Authority received 4939 *L. monocytogenes* isolates from foods and food production plants (average 449/year, range 284–572) from 23 local official food control laboratories. Isolated strains represented 3353 food or food production plant samples (average 305/year, range 202–394). In 2011–2021, 3.1% (104/3353) of food samples (3–18 samples per year) contained more than 100 colony-forming units (CFU)/g of *L. monocytogenes.*

In total, 626 (13%) *L. monocytogenes* isolates from foods or food production plants were genotyped in 2011–2021. During 2011–2017, 105 different PFGE AscI profiles were detected, type 96 being the most common (90/439, 21%). In 2017–2021, when WGS was used as the typing method, 36 different 7-gene MLST types were identified among the strains typed.

In 2012–2014, the Finnish Food Authority received results of 793 sliced, heat-treated, or cold-smoked ready-to-eat meat product samples from local official food control laboratories as part of an official survey. Of the products, 94% were manufactured in Finland, and 47% of the food samples contained only domestic meat. Of the analysed food samples, 82% (652/793) were stored in packaging gas and 16% (126/793) were vacuum-packed. In 11% of the samples, the temperature of the product in retail was above the statutory limit of 6.0 degrees Celsius. At the end of shelf-life, *L. monocytogenes* was detected in 10 (1.3%) samples. In nine of those samples, the count of *L. monocytogenes* was 10 CFU/g or below. One bacterial count result was missing.

In 2015–2016, the Finnish Food Authority received results of 398 sliced cheese samples from local official food control laboratories. The samples were from domestic and foreign, packed, sliced cheeses in retail. *L. monocytogenes* was not found.

### Comparison of food and patient isolates

There were 16 mutual PFGE AscI profiles in patient samples in 2011–2015 and in food and food production plant samples in 2011–2016 (Table 1). Of all PFGE AscI typed isolates, 40% (113/282) of patient strains and 41% (182/439) of food and food production plant strains belonged to these 16 mutual profiles. PFGE profiles 21, 70, and 72 were found only in fish, profile 44 only in meat products, and profiles 42 and 68 only in vegetable products. The three most common mutual PFGE AscI profiles, 14, 62 and 96, were found mainly in raw fish or fishery products. The majority (63%, 69/109) of listeria contaminated fish and fishery products were likely to be consumed uncooked (the strain was isolated from cured or cold-smoked salmon).

In 2015–2021, 687 strains of *L. monocytogenes* were sequenced. These originated from patients (490), food items (184), and samples from food production environments (13). The MLST type of seven patient samples and two food samples could not be determined. The strains showed 49 MLST types, 16 of which contained nine or more patient isolates (Table 2). These 16 most common MLST-types included 408/490 (83%) of patient isolates and 23 clusters defined by cgMLST, each containing five or more patient isolates. Of all 490 patient isolates analysed, 262 (53%) fell into these clusters. In 12 of the clusters, also food or environmental isolates were detected.

## Discussion

In Finland, a slight increase in the incidence of invasive listeriosis has been seen in the past 10 years. A similar trend has been seen in Sweden [19]. At the EU level, the overall trend for listeriosis has been stable during 2017–2021 [5]. In our study, listeriosis was most common in age groups over 75 years old. Additionally, in other EU countries, most cases have been reported among the elderly, which is partly explained by the ageing population and chronic age-related illnesses [5]. In Finland, the proportion of those aged over 64 years is expected to rise from 23% in 2021 to 33% in 2070 [20]. The higher number of people more susceptible to listeriosis may lead to increases in listeriosis cases if appropriate control measures are not taken.

In Finland, listeriosis in pregnant women is rare. In 2011–2021, 4% of notified cases in Finland were reported as materno-foetal, all of which were sporadic cases. In England and Wales in 2020, 20% of all reported listeria cases were pregnancy-associated [21]. In the literature, one in seven cases of listeriosis is estimated to occur in pregnant women [1]. Awareness of listeria has been shown to reduce the consumption of high-risk foods; awareness was higher in mothers older than 25 years when compared to younger mothers [22]. In Finland in 2020, the mean age of birth givers was 31 years, with only 1.2% of all birth givers being under 20 years of age [23]. Food recommendations for families with children were published in 2016 by the National Nutrition Council and THL and updated in 2019 [24]. Pregnant women are advised to eat fish products only properly heated and to avoid eating sushi, roe, and foods containing raw fish to avoid the risk of listeriosis. In addition, unheated meat products, raw or unpasteurized milk, cheese made from unpasteurized milk, soft cheeses, frozen vegetables, and processed foods are recommended to be avoided or consumed only properly heated. Unheated meat cold cuts are deemed safe if consumed well before the use-by date. The avoidable risk foods differ somewhat between countries, especially regarding fish products, fruits, and vegetables [25]. In Finland, the recommendations are communicated to mothers at the maternity clinics, which are free of charge for all mothers. According to the Medical Birth Register, they are widely utilized with only 0.2–0.3% of the mothers not attending them during pregnancy [26]. Thus, it can be reasoned that Finnish mothers are probably well aware of the risk of listeriosis.

The case fatality rate in Finland was slightly higher (22%) than the EU average (14% in 2021 [5]). However, in the EU data, the outcome was reported for only 65% of the confirmed cases. In the literature, mortality rates of 20–30% are described [3, 27]. Most of the interviewed cases in our study were immunocompromised due to an underlying illness or medication used. Immunosuppressing treatments and conditions, such as liver and renal diseases, and diabetes mellitus, are known risk factors for invasive listeriosis [3]. According to a population-based Finnish health survey from 2017, 18% of males over 65 years of age had coronary heart disease, 25% had diabetes, and 7% had chronic obstructive pulmonary disease while in women these illnesses were less common (12%, 18%, and 3%, respectively) [28]. Compared to these survey data, the listeriosis cases in our study had more comorbidities than the general population.

Despite being immunocompromised, the cases had often consumed high-risk foods, such as ready-to-eat meat products, cured and cold- or hot-smoked fish, and salami or other sliced sausages. Over half of the cases did not have knowledge of high-risk foods for listeriosis prior to infection. In addition, one third of cases checked their fridge’s temperature monthly or less often; one fifth reported throwing out outdated products monthly or less often. This suggests that recommendations about high-risk foods and proper food storage should be highlighted also to other risk groups than pregnant women. THL and the Finnish Food Authority have produced a leaflet on listeriosis for doctors to distribute to patients [29]. However, more proactive communication about listeriosis to risk groups is needed. People at higher risk of listeriosis can reduce the risk by thoroughly cooking food, heating ready-to-eat foods till they are steaming hot, and avoiding outdated products as well as risk foods that are not properly heated. In addition, high-risk foods should not be served for those unable to evaluate the risk of listeriosis themselves such as the elderly suffering from forgetfulness or persons living in institutional care. Since *L. monocytogenes* is capable of growth in refrigeration temperatures and growth is faster in higher temperatures [30], it is important to maintain a low temperature in the fridge and consume ready-to-eat products well before the use-by date if not heated. The fridge temperature should be checked regularly, and the proper temperature (<+6 °C; <+3 °C for fish products [29]) should be highlighted to consumers. Fridge thermometers could be provided for the elderly along with stickers for the fridge door reminding them of the correct temperature.

Half of the cases in our study had used medication to treat gastric acidity. Use of proton pump inhibitors (PPI) and other medications that neutralize or inhibit gastric acid are considered to increase the risk of listeriosis, possibly because a low gastric pH is a natural defence against listeria [31, 32]. The same applies also for other enteric pathogens, such as *Salmonella* and *Campylobacter* [32]. The potential risks of PPI usage should be better communicated to the public, since their use has increased in recent decades and is more common in the elderly [33, 34], who are also more likely to have immunocompromising conditions.

In over one third of the cases, the patient’s spouse or other relative answered the questionnaire due to the frail condition or forgetfulness of the patient. The incubation period of listeriosis varies by clinical manifestation, but the median is estimated to be 11 days with most cases occurring within four weeks [35]. Old age, forgetfulness, and second-hand information as well as the long incubation period of listeriosis can lead to a recall bias and affect the exposure results gleaned from the interviews. To minimize the recall bias, interviews should be performed as soon as possible after the onset of illness. Using consumer food purchase data helps to solve outbreaks [36] and has been used also in Finland.

The serogroup IIa accounted for most of the listeriosis cases followed by IVb. According to the literature, these serotypes account for most human infections [3]. In 2011–2014, serotyping and PFGE were used to genotype the patient samples in Finland. In 2015, WGS was introduced for listeria confirmation and typing in parallel with PFGE. We identified three clusters that were concordant with both methods. However, the methods were not completely comparable, since two PFGE types were included in two MLST types. Since 2016, WGS has been the sole typing method for patient samples in Finland and its usage has enhanced the detection of listeria clusters. Compared to PFGE, cgMLST provides better discrimination and accuracy in typing listeria strains [37]. By using WGS, in 2015–2021 we identified 23 clusters with five or more patient isolates, many of which persisted for years. Since 2018, the Finnish Food Authority started typing food isolates with a similar method to that used for patient isolates, enabling the comparison of listeria strains. We found matching food isolates that gave microbiological evidence to support epidemiological findings in listeriosis outbreak investigations. This indicates that tracing the source of infection is possible if data from patient interviews, production plants, and product distribution are available. Interview data can be used to direct the typing of food isolates stored in the strain collection. Similarly, information on products in which matching listeria isolates have been found can be used to adjust questionnaires for interviews.

In the eight listeriosis outbreaks that could be solved with both epidemiological and microbiological evidence since starting the listeriosis case interviews, 133 people fell ill. The causative food items belonged to fish products (cured and cold-smoked salmon, frozen rainbow trout fillet), fresh or frozen vegetables (frozen corn, chopped iceberg lettuce, cabbage salads), and meat products (turkey cold cuts, frankfurter, meat jelly). The outbreaks were long-lasting, with the duration varying from three to seven years. In 2016–2018, a widespread outbreak affecting five European countries was caused by frozen corn [38]. This outbreak was detected, and the frozen corn as the outbreak source was identified, in Finland.

The overall occurrence of listeria in food products in Finland is currently unknown. A small proportion of food samples investigated have contained more than the regulatory limit of 100 CFU/g of *L. monocytogenes.* In 2010, the Finnish Food Authority received 323 food *L. monocytogenes* isolates of which the majority were genotyped [12]. Since 2014, only strains suspected to be linked to outbreaks have been typed. In the surveys conducted by the Finnish Food Authority, listeria was not found in sliced cheese samples in 2015–2016. In 2012–2014, listeria was rarely detected in sliced, heat-treated, or cold-smoked ready-to-eat meat product samples tested. Aalto-Araneda et al. investigated the occurrence of listeria in ready-to-eat vacuum-packed cured and cold-smoked fish products in 2014–2015 and found 4.2% of the sampled packages to be positive for *L. monocytogenes* [39]. Since 2016, no national sampling surveys have been conducted. As the indication for typing strains isolated from food is an outbreak investigation, the occurrence of different MLST types and their genetic relationships to one another in contaminated food products and process environments in Finland has yet to be investigated. Typing food isolates is crucial to both outbreak investigations and for controlling the risk of listeriosis in the food industry.

## Conclusions

In Finland, the notification rate for listeriosis is higher than the EU average, and the trend is slightly increasing (from 0.8/100000 in 2011 to 1.3/100000 in 2021), probably due to the ageing population and age-related illnesses affecting the immune system. Pregnancy-related listeriosis is rare, due to effective maternity clinic counselling. Recommendations about high-risk foods for listeriosis should be better communicated to other risk groups, as well as to relatives and people taking care of the elderly. Several listeriosis outbreaks have been detected and food sources identified since ongoing patient interviews and WGS were introduced. Patient interviews and testing and typing listeria isolates in foods and comparing them to patient isolates are crucial for solving outbreaks and determining measures to control invasive listeriosis in Finland.

## Data Availability

The raw/processed data analysed in the study cannot be shared due to European General Data Protection Regulation.

## Supplementary material

For supplementary material accompanying this paper visit http://doi.org/10.1017/S0950268823001073. click here to view supplementary material
