# Supplementary material for: Invasive listeriosis in Finland: surveillance and cluster investigations, 2011–2021
Source: Epidemiol Infect. 2023 Jul 10;151:e118. doi: 10.1017/S0950268823001073 (PMC10468812; doi:10.1017/S0950268823001073)
Supplement: Supplementary file 1 [file S0950268823001073sup001.docx]

**Supplementary Table 1**. The *Listeria monocytogenes* MLST types detected from patients and case fatality rate, in Finland in 2015–2021.

| MLST  (Serotype) | Number of patients* | Case fatality rate, %^†^ |
| --- | --- | --- |
| ST-7  (IIa) | 52 | 21 |
| ST-6  (IVb) | 48 | 13 |
| ST-9  (IIc) | 41 | 15 |
| ST-451  (IIa) | 39 | 26 |
| ST-8  (IIa) | 38 | 24 |
| ST-37  (IIa) | 37 | 24 |
| ST-155  (IIa) | 24 | 33 |
| ST-1  (IVb) | 23 | 4 |
| ST-120  (IIa) | 22 | 14 |
| ST-18  (IIa) | 16 | 25 |
| ST-4  (IVb) | 14 | 21 |
| ST-206  (IIa) | 12 | 33 |
| ST-14  (IIa) | 11 | 36 |
| ST-91  (IIa) | 11 | 27 |
| ST-2408  (IVb) | 11 | 27 |
| ST-391  (IIa) | 9 | 33 |
| ST-124 | 6 | 0 |
| ST-19 | 5 | 40 |
| ST-101 | 5 | 20 |
| ST-2 | 4 | 25 |
| ST-20 | 4 | 25 |
| ST-21 | 4 | 25 |
| ST-400 | 4 | 0 |
| ST-26 | 3 | 0 |
| ST-29 | 3 | 0 |
| ST-59 | 3 | 1 |
| ST-87 | 3 | 0 |
| ST-121 | 3 | 1 |
| ST-173 | 2 | 100 |
| ST-177 | 2 | 0 |
| ST-321 | 2 | 0 |
| ST-398 | 2 | 100 |
| ST-403 | 2 | 0 |
| ST-814 | 2 | 0 |
| ST-1247 | 2 | 50 |
| ST-11 | 1 | 0 |
| ST-31 | 1 | 100 |
| ST-77 | 1 | 0 |
| ST-200 | 1 | 0 |
| ST-219 | 1 | 0 |
| ST-220 | 1 | 0 |
| ST-388 | 1 | 0 |
| ST-394 | 1 | 0 |
| ST-416 | 1 | 100 |
| ST-570 | 1 | 0 |
| ST-849 | 1 | 0 |
| ST-994 | 1 | 100 |
| ST-1344 | 1 | 0 |
| ST-2488 | 1 | 0 |

*One isolate per patient

^†^Within 30 days of sampling
